# Polymorphisms in three genes are associated with hemorrhagic stroke

**DOI:** 10.1002/brb3.395

**Published:** 2015-10-01

**Authors:** Wenpeng Liu, Shichao Ge, Yan Liu, Can Wei, Yunlong Ding, Aimin Chen, Qiyao Wu, Yuqing Zhang

**Affiliations:** ^1^ Department of Internal Medicine of NeurologyPeople's Hospital of Jingjiang CityJingjiang214500Jiangsu ProvinceChina; ^2^ Department of Research and DevelopmentShanghai Benegene Biotechnology Co., Ltd.Shanghai201114China

**Keywords:** Association, genotype, *Golgb1* gene, hemorrhagic stroke, *RAGE* gene, single nucleotide polymorphisms, *TNFRSF11B* gene

## Abstract

**Background:**

Multiligand receptor for advanced glycation end products (RAGE), osteoprotegerin, and *Golgb1* genes may be implicated in atherosclerosis and vascular diseases. Single nucleotide polymorphisms (SNPs) rs1035798 in *RAGE* gene, rs2073617 and rs2073618 in *TNFRSF11B*, and rs3732410 in *Golgb1* will be investigated on whether there is an association with hemorrhagic stroke (HS) in Chinese population.

**Methods:**

A total of 600 subjects including 199 HS patients and 401 controls were assayed. These samples were divided into two groups: the ≤50 year and >50 year groups. Genotyping of SNPs was determined using the SEQUENOM MassARRAY matrix‐assisted laser desorption ionization–time‐of‐flight–mass spectrometry. The association between genotype and HS risk was evaluated by computing the odds ratio (OR) and 95% confidence interval (CI) with multivariate unconditional logistic regression analyses.

**Results:**

Our data showed that in the ≤50 year group, the rs1035798 major allele homozygote C/C in *RAGE* gene was associated with an increased risk of HS, while *Golgb1* rs3732410 minor allele homozygote G/G was associated with a decreased risk of HS. In the >50 year group, the major allele homozygote G/G of rs2073618 was found to be associated with an increased risk of HS.

**Conclusions:**

The polymorphisms rs1035798 of *RAGE* gene, rs2073618 of *TNFRSF11B*, and rs3732410 of *Golgb1* might be involved in the risk of HS at different stage of ages.

## Introduction

Stroke is the second leading cause of death in population more than 60 years old and the fifth leading cause of death in people aged 15–59 years old in the world (Johnston et al. 2009). In China, the annual stroke mortality rate has exceeded heart disease to become the leading cause of death and adult disability (Liu et al. 2011). Stroke is divided into two types: ischemic and hemorrhagic stroke, with the latter including intracerebral hemorrhage and subarachnoid. Compared to white populations of European origin, Chinese populations have a higher incidence of stroke overall, a higher proportion of hemorrhagic stroke (HS) (Tsai et al. 2013). The previous studies suggested that at least 30% of strokes in China were HS (Hong et al. 1994). The risk factor profiles and prevention strategies are different for ischemic and hemorrhagic stroke (Leppala et al. 1999).

The multiligand receptor for advanced glycation end products (RAGE, alias AGER) contributes to the pathogenesis of vascular disease (Kalea et al. 2009; Olsson and Jood 2013). The upregulation of *RAGE* expression was found in human atherosclerotic plaques and aortic vessels (Ritthaler et al. 1995; Cipollone et al. 2003). Variants in the *RAGE* gene, such as rs1800625, rs1800624, and rs2070600 polymorphisms, had been shown to be associated with diabetic atherosclerosis (Pettersson‐Fernholm et al. 2003). In one case–control study, genetic variation rs1035798 SNP in the *RAGE* gene was observed to be associated with the subtype of small‐vessel disease (SVD), but not with overall ischemic stroke (IS) (Olsson and Jood 2013). No study reported the association between rs1035798 SNP and HS.

Osteoprotegerin (OPG) is a glycoprotein, serving as a soluble decoy receptor for two members of the tumor necrosis factor receptor superfamily: RANKL and TRAIL (receptor activator of nuclear factor‐*κ*B ligand and tumor necrosis factor–related apoptosis‐inducing ligand) (Bord et al. 2004). OPG inhibits osteoclastogenesis and function of differentiated osteoclasts, thereby preventing bone resorption (Biscetti et al. 2013). Several lines of evidence supported that *TNFRSF11B* gene product OPG is not only a marker but also a mediator of vascular disease (Jono et al. 2002; Schoppet et al. 2003). Serum OPG concentrations have been found to correlate with progressive atherosclerosis and cardiovascular diseases (Kiechl et al. 2004; Ziegler et al. 2005). Although several studies have investigated *OPG* gene involved in ischemic stroke (Dichgans 2007; Guldiken et al. 2007; Biscetti et al. 2013), studies on the relation between polymorphisms of *OPG* and HS are still less.

*Golgb1* gene encodes the coat protein 1 (COP1) vesicle tethering factor, Giantin, which is responsible for the phenotypic characteristics including osteochondrodysplasia and plays a pivotal role in multiple aspects of chondrogenesis (Katayama et al. 2011). The *Golgb1* rs3732410 mutation is a naturally occurring variant and associated with protection from ischemic stroke (Flanagan et al. 2013). However, no data have been recorded on the relationship between this site and HS.

In the present study, the abovementioned SNPs including rs1035798 in *RAGE* gene, rs2073617 and rs2073618 in *TNFRSF11B*, and rs3732410 in *Golgb1* were investigated on the association with HS in Chinese population for the first time.

## Materials and Methods

### Study subjects

Study subjects were recruited from consecutive hemorrhagic stroke patients and unrelated age‐matched healthy controls from the People's Hospital of Jingjiang City, Jiangsu Province, China, between February 2012 and April 2014. Hemorrhagic stroke included cerebral hemorrhage and subarachnoid hemorrhage (Zhang et al. 2013). Patients with hemorrhage due to trauma, tumor, vascular malformation, and coagulopathy were excluded. Control subjects were recruited from the health examination department of the hospital. These subjects had no clinical or radiological evidence of stroke and other neurological diseases. All subjects were of Han origin and lived roughly within the same geographic region. Sex, age, body mass index (BMI), total cholesterol (TC), triglycerides (TG), and fasting glucose (FG) were collected on entry into the study. Potential vascular risk factors were evaluated, including hypertension, diabetes mellitus, drinking, and smoking. Hypertension was defined as systolic blood pressure ≥140 mmHg and/or diastolic pressure ≥90 mmHg according to the World Health Organization criteria. Diabetes mellitus was defined as fasting plasma glucose ≥7.0 mmol/L. Written informed consent was obtained from all subjects before participation. The study protocol was approved by the Research Ethics Committee of the People's Hospital of Jingjiang City, Jiangsu Province, China.

### SNPs selection and genotyping

Single nucleotide polymorphisms rs1035798 in *RAGE*, rs2073617 and rs2073618 in *TNFRSF11B*, and rs3732410 in *Golgb1* were selected according to the previously published papers (Biscetti et al. 2013; Flanagan et al. 2013; Olsson and Jood 2013). Genomic DNA was extracted from whole blood using the AxyPrep Blood Genomic DNA Miniprep kit (Axygen Biosciences, Union City, CA), and the SNPs were genotyped using the SEQUENOM MassARRAY matrix‐assisted laser desorption ionization–time‐of‐flight–mass spectrometry platform (SEQUENOM, San Diego, CA). Primers for polymerase chain reaction (PCR) were designed using Assay Designer software version 3.0 (SEQUENOM) and synthesized by Sangon Biotech (Shanghai, China), and the primer sequences were showed in Table 1. PCR conditions were as follows: denaturation at 94°C for 15 min, followed by 45 cycles of 20 sec at 94°C, 30 sec at 56°C, 1 min at 72°C, and a final extension of 3 min at 72°C. The final primary PCR reaction mix was treated with shrimp alkaline phosphatase to dephosphorylate unincorporated dNTPs. The iPLEX primer extension reaction was further performed with PCR conditions: denaturation at 94°C for 5 sec, followed by 40 cycles of 94°C for 5 sec, and 52°C for 5 sec and 80°C for 5 sec (5 cycles), 72°C for 3 min, and holding at 4°C. PCR products were purified with resin with procedures followed the iPLEX kit standard protocol (SEQUENOM). The purified extension products were spotted onto a 384‐well specroChip (Affymetrix, USA) by using MassARRAY Nanodispenser and determined with the matrix‐assisted laser desorption ionization–time‐of‐flight–mass spectrometer (MALDI‐TOF‐MS). Genotype analysis was performed in real time with MassARRAY RT software version 3.1 and analyzed by using the MassARRAY Typer software version 4.0 (SEQUENOM).

**Table 1:** Oligonucleotide sequences used for genotyping

Genes	SNPs	Primers	Sequences
*RAGE*	rs1035798	First	ACGTTGGATGTTCCCAGGAATCTCTGAAGG
		Second	ACGTTGGATGTGACTTCCACTGGCCTCATT
		Extension	CCTCTTAGTCTCCACAC
*TNFRSF11B*	rs2073617	First	ACGTTGGATGTTTTTAGGAAGCTTGGGCGG
		Second	ACGTTGGATGTTTCCGCCCCAGCCCTGAAA
		Extension	GTTGCCGCCCCAGCCCTGAAAGCGTTAA
	rs2073618	First	ACGTTGGATGCCAGGGACTTACCACGAGC
		Second	ACGTTGGATGTCCAAGCCCCTGAGGTTTC
		Extension	GCGGTTTCCGGGGACCACAATGAACAA
*Golgb1*	rs3732410	First	ACGTTGGATGCCTTGCTTTGCTCATCAAAC
		Second	ACGTTGGATGAGAAAGACATCTCAGGGAGG
		Extension	GGGAAGCAACAGAAAGATGACT

### Statistical analysis

Statistical analyses were performed using SPSS 13.0 for Windows (SPSS Inc., Chicago, IL). Hardy–Weinberg equilibrium (HWE) was carried out for all SNPs of samples. The hemorrhagic stroke cases and controls were compared using the *χ* ^2^ test, and *P* < 0.001 was considered statistically different. The association between SNPs and hemorrhagic stroke risk was analyzed by computing the odds ratio (OR) and 95% confidence interval (CI) with multivariate unconditional logistic regression analyses, and a two‐sided *P* < 0.05 was considered statistically significant.

## Results

### Clinical characteristics of HS patients and controls

A total of 199 HS patients and 401 controls were recruited in the study. These samples were divided into two groups: the ≤50 year and the >50 year groups by age stratification. In the ≤50 year group, in terms of age, gender (men %), body mass index (BMI), triglycerides, and hypertension, there are significant differences between the patients and controls (*P* < 0.001), while no difference between the patients and controls on total cholesterol, fasting glucose, and diabetes was found. In the >50 years group, there are significant differences between the patients and controls in terms of age, gender (men %), body mass index (BMI), fasting glucose, hypertension, and diabetes (*P* < 0.001), while no difference between the HS patients and controls on total cholesterol and triglycerides was found. There are significant differences in certain vascular risk factors between HS patients and control subjects (Obach et al. 2001; Yoshida et al. 2010). Characteristics of the patients and controls are summarized in Table 2.

**Table 2:** Characteristics of HS patients and controls

Characteristic	≤50 year group			>50 year group		
	Cases	Control	*P* (trend)	Case	Control	*P* (trend)
No. of subjects	33	255		166	146	
Age (years)	46 ± 3.5	34 ± 9.3	**<0.001**	67 ± 8.4	61 ± 5.9	**<0.001**
Gender (% men)	69.7	62.4	**<0.001**	63.3	58.2	**<0.001**
BMI (kg/m^2^)	27.1 ± 1.8	23.9 ± 1.5	**<0.001**	26.9 ± 2.1	23.8 ± 1.5	**<0.001**
TG (mmol/L)	2.1 ± 1.53	1.5 ± 0.89	**<0.001**	1.7 ± 0.97	1.6 ± 0.89	0.390
TC (mmol/L)	5.0 ± 0.88	4.7 ± 0.81	0.027	4.8 ± 0.98	4.8 ± 0.84	0.674
FG (mmol/L)	5.6 ± 0.93	5.4 ± 1.30	0.289	6.2 ± 2.45	5.4 ± 1.10	**<0.001**
Hypertension (%)	90.9	2.0	**<0.001**	92.8	21.2	**<0.001**
Diabetes (%)	15.2	3.5	0.115	13.9	2.7	**<0.001**

BMI, body mass index; TG, triglycerides; TC, total cholesterol and FG, fasting glucose.

Bold numbers indicate that the differences were statistically significant between the two groups.

For four SNPs, samples were within Hardy–Weinberg equilibrium (HWE) (*P* = 0.25515 for *RAGE* rs1035798, 0.24144 for *TNFRSF11B* rs2073617, 0.25587 for *TNFRSF11B* rs2073618, and 0.36587 for *Golgb1* rs3732410, respectively), suggesting that these SNPs were not in linkage disequilibrium.

### Allele frequencies of SNPs between the HS patients and controls

For four SNPs in the ≤50 year group, allele frequency of *RAGE* rs1035798 was 4.5% (T) and 95.5% (C) in HS patients, 13.7% (T) and 86.3% (C) in control; allele frequency of *TNFRSF11B* rs2073617 was 59.1% (T) and 40.9% (C) in HS patients, 62% (T) and 38% (C) in control; allele frequency of *TNFRSF11B* rs2073618 was 33.3% (C) and 66.7% (G) in HS patients, 75.5% (G) and 24.5% (C) in controls; allele frequency of *Golgb1* rs3732410 was 59.1% (A) and 40.9% (G) in HS patients, 54.1% (A) and 45.9% (G) in controls. Except that there was a significant difference in allele frequencies of *RAGE* rs1035798 between the HS patients and the control subjects (*P* = 0.0349), no statistical differences were found in allele frequencies of the remaining three SNPs (*P* > 0.05) (Table 3). For all four SNPs in the >50 year group, the differences were found not statistically significant between the HS patients and the control subjects.

**Table 3:** Allele frequency of SNPs in HS patients and controls

Gene SNPs	Allele	≤50 year group					>50 year group				
		Cases *N* (%)	Control *N* (%)	*χ* ^2^	*P* value	OR (95% CI)	Cases *N* (%)	Control *N* (%)	*χ* ^2^	*P* value	OR (95% CI)
*RAGE*											
rs1035798	T	3 (0.045)	70 (0.137)				37 (0.111)	37 (0.127)			
	C	63 (0.955)	440 (0.863)	4.4497	**0.0349**	0.299 (0.091–0.979)	295 (0.889)	255 (0.873)	0.3464	0.5562	0.864 (0.532–1.405)
*TNFRSF11B*											
rs2073617	T	39 (0.591)	315 (0.620)				213 (0.645)	187 (0.640)			
	C	27 (0.409)	193 (0.380)	0.2103	0.6466	0.885 (0.525–1.492)	117 (0.355)	105 (0.360)	0.0172	0.8958	1.022 (0.736–1.420)1
rs2073618	C	22 (0.333)	125 (0.245)				70 (0.211)	80 (0.276)			
	G	44 (0.667)	385 (0.755)	2.3936	0.1218	1.54 (0.888–2.670)	262 (0.789)	210 (0.724)	3.5758	0.0586	0.701 (0.485–1.014)
*Golgb1*											
rs3732410	A	39 (0.591)	276 (0.541)				170 (0.512)	164 (0.562)			
	G	27 (0.409)	234 (0.459)	0.5833	0.445	1.225 (0.728–2.061)	162 (0.488)	128 (0.438)	1.5362	0.2152	0.819 (0.597–1.123)

Bold numbers indicate that the differences were statistically significant between the two groups.

### Genotype distribution of SNPs between HS patients and controls

Genotype distributions of SNPs were analyzed between the cases and controls. In the ≤50 year group, the polymorphisms rs1035798 major allele homozygote C/C in *RAGE* gene were associated with more than threefold risk of HS (*χ* ^2^ = 4.3568, *P* = 0.0369; OR = 3.421, 95% CI = 1.010–11.585), and rs3732410 minor allele homozygote G/G in *Golgb1* gene were associated with one‐fourth fold risk of HS (*χ* ^2^ = 4.0998, *P* = 0.0429; OR = 0.2459, 95% CI = 0.057–1.06) (Table 4). There was no significant difference in genotype frequencies of *TNFRSF11B* rs2073617 and rs2073618 between the HS patients and control subjects (*P* > 0.05). In the >50 year group, the genotype frequency of *TNFRSF11B* rs2073618 was 5.4% (CC), 63.3% (GG), and 31.3% (CG) in HS patients, 6.9% (CC), 51.7% (GG), and 41.4% (CG) in controls. The major allele homozygote G/G of rs2073618 was found to be associated with an increased risk of HS (*χ* ^2^ = 4.2196, *P* = 0.04; OR = 1.607, 95% CI = 1.021–2.528). No association was found for other SNPs between the HS patients and controls (*P* > 0.05). These results indicated that rs1035798 major allele homozygote C/C in *RAGE* gene were associated with an increased risk of HS, while *Golgb1* rs3732410 minor allele homozygote G/G was associated with a decreased risk of HS in younger ages; and *TNFRSF11B* rs2073618 major allele homozygote G/G was associated with a increased risk of HS in older ages in Chinese population.

**Table 4:** Genotype distributions of SNPs in HS patients and controls

Gene SNPs	Genotype	≤50 year group					>50 year group				
		Cases *N* (%)	Control *N* (%)	*χ* ^2^	*P* value	OR (95% CI)	Cases *N* (%)	Control *N* (%)	*χ* ^2^	*P* value	OR (95% CI)
*RAGE*											
rs1035798	CC	30 (0.909)	190 (0.745)				131 (0.789)	114 (0.781)			
	TT	0 (0.000)	5 (0.020)				2 (0.012)	5 (0.034)			
	CT	3 (0.091)	60 (0.235)	4.4603	0.1075		33 (0.199)	27 (0.185)	1.7906	0.4085	
	TT+CT	3 (0.091)	65 (0.255)	4.3568	**0.0369**	3.421 (1.010–11.585)	35 (0.211)	32 (0.219)	0.032	0.858	1.051 (0.612–1.805)
*TNFRSF11B*											
rs2073617	CC	5 (0.152)	31 (0.122)				26 (0.158)	13 (0.089)			
	TT	11 (0.333)	92 (0.362)				74 (0.448)	54 (0.370)			
	CT	17 (0.515)	131 (0.516)	0.27	0.8737		65 (0.394)	79 (0.541)	7.6874	0.0214	
	CC+CT	22 (0.667)	162 (0.638)	0.1058	0.745	0.88 (0.409–1.897)	91 (0.552)	92 (0.630)	1.9771	0.1597	1.385 (0.879–2.184)
rs2073618	CC	4 (0.121)	19 (0.075)				9 (0.054)	10 (0.069)			
	GG	15 (0.455)	149 (0.584)				105 (0.633)	75 (0.517)			
	CG	14 (0.424)	87 (0.341)	2.2369	0.3268		52 (0.313)	60 (0.414)	4.2253	0.1209	
	CC+CG	18 (0.545)	106 (0.416)	2.0069	0.1566	0.593 (0.286–1.229)	61 (0.367)	70 (0.483)	4.2196	**0.04**	1.607 (1.021–2.528)
*Golgb1*											
rs3732410	AA	8 (0.242)	74 (0.290)				45 (0.271)	43 (0.295)			
	AG	23 (0.697)	128 (0.502)				80 (0.482)	78 (0.534)			
	GG	2 (0.061)	53 (0.208)	5.6703	0.0587		41 (0.247)	25 (0.171)	2.6785	0.262	
	AG+AA	31 (0.939)	202 (0.792)	4.0998	**0.0429**	0.2459 (0.057–1.060)	125 (0.753)	121 (0.829)	2.6728	0.1021	1.588 (0.910–2.770)

Bold numbers indicate that the differences were statistically significant between the two groups.

## Discussion

In the current study, we investigated the association between genetic variants in *RAGE*,* TNFRSF11B*, and *Golgb1* genes and risk of HS. The results showed that rs1035798 C/C in *RAGE* gene were strongly associated with an increased risk of HS, while the *Golgb1* rs3732410 polymorphism was associated with a reduced risk of HS in younger group (≤50 years old), and the *TNFRSF11B* rs2073618 was associated with an increased the risk of the disease in elder group (>50 years old). This suggests that *RAGE* rs1035798 and *Golgb1* rs3732410 variants play a role at a younger age and *TNFRSF11B* rs2073618 variant exhibited a role in HS occurrence at an elderly age. To the best of our knowledge, this is the first report to demonstrate an association of *RAGE* rs1035798, *Golgb1* rs3732410, and *TNFRSF11B* rs2073618 SNPs with HS risk. However, *TNFRSF11B* rs2073617 was not associated with HS, irrespective of age factor.

The human *RAGE* gene is located in the major histocompatibility complex (MHC) class III region on chromosome 6p21.3, consisting of 11 exons, a 3′ UTR (untranslated region) and a 5′ flanking region which overlaps the 3′ UTR of the *PBX2* (Zee et al. 2006; Kalea et al. 2009). Genetic studies have identified that approximately 30 polymorphisms occur in the *RAGE* gene. The upregulation of *RAGE* gene expression is a hallmark in vascular disease and therefore genetic variants affecting *RAGE* mRNA or protein levels may therefore be important disease markers (Kalea et al. 2009). In sex‐specific analyses, association to overall IS in women but not in men were observed for rs1035798 (OR = 1.36; 95% CI = 1.05–1.76) and rs1800684 (OR = 0.53, 95% CI = 0.36–0.77) (Olsson and Jood 2013). Further analysis revealed that rs1035798 showed significant association with the subtype of SVD after correction for multiple testing (OR = 1.56; 95% CI = 1.16–2.09), but no association with overall IS. Interestingly, in this study, we found the polymorphism rs1035798 was strongly associated with an increased risk of HS (OR = 3.421, 95% CI = 1.010–11.585) in the ≤50 year group, but not in elder group (>50 years old). Anyway, this is the first time to reveal the correlation between this SNP with HS in Chinese population.

Osteoprotegerin, encoded by *TNFRSF11B* gene, is a member of the tumor necrosis factor receptor superfamily of cytokine that regulates osteoclastogenesis (Simonet et al. 1997). Cells within the cardiovascular system, such as arterial smooth muscle cells, endothelial cells, and megakaryocytes secreted OPG into the circulation (Malyankar et al. 2000; Bord et al. 2004). OPG‐G1181C (rs2073618) is the only polymorphism in a coding region of *TNFRSF11B*, while OPG‐T950C (rs2073617) is located in its promoter region (Tsai et al. 2013). This G/C variation results in lysine/asparagine change in the single peptide of OPG, which may have an effect on intracellular trafficking or export efficiency of the final protein and local concentration of OPG in the vessel wall. Aside from being an important regulating molecule in bone formation and resorption (Ziegler et al. 2005; Strand et al. 2007), OPG also served as a vascular calcification inhibitor (Clancy et al. 2006). Studies showed that OPG was an independent predictor of cardiovascular disease (Nybo and Rasmussen 2008; Omland et al. 2008). The previous study reported that rs2073617 C/C and rs2073618 C/C variant genotypes of the *OPG* gene were significantly and independently associated with the increased risk of ischemic stroke in an Italian population with diabetic patients (Biscetti et al. 2013). Carriers of the OPG‐1181 (rs2073618) C/C genotype had a significantly increased risk of intracerebral hemorrhage (ICH), but no associations were found between C/C genotype and ischemic stroke, and nor between the OPG‐T950C (rs2073617) and stroke subtypes (Tsai et al. 2013), which is consistent with our observation for this SNP. The current study showed the rs2073618 G/G variant genotype was associated with hemorrhagic stroke in Chinese population after 50 years, but not with the younger population of ≤50 years old.

*Golgb1* gene, located on chromosome 11, encodes Giantin (Katayama et al. 2011). It has been reported that *Golgb1* gene is expressed in cultured chondrocytes (Johansen et al. 2001). Giantin is a Golgi apparatus–associated protein and presents on the Golgi membrane and coat protein 1 (COP1) vesicles. The best characterized function of Giantin is to tether COP1 vesicles to the Golgi apparatus, allowing bidirectional cargo transport through the Golgi stack (Malhotra et al. 1989; Orci et al. 1997). Genome‐wide association studies revealed that one mutation in *Golgb1* Y1212C (rs3732410) appeared to have a dominant effect: the overall significance of this variant with a reduced risk of ischemic stroke in all sickle cell anemia (SCA) patients with stroke against control SCA patients (OR = 0.27; 95% CI = 0.14–0.52) (Katayama et al. 2011). Cerebrovascular disease is perhaps the most devastating complication with SCA for younger patients. This study observed that the *Golgb1* rs3732410 minor allele homozygote G/G was strongly associated with a decreased risk of HS (OR = 0.2459, 95% CI = 0.057–1.06; Table 4) in the ≤50 year group. These suggested that *Golgb1* rs3732410 variant have an important role in ischemic or hemorrhagic stroke in younger patients.

## Conclusion

The current study demonstrated that *RAGE* rs1035798 and *Golgb1* rs3732410 appeared to be strongly associated with an increased and a decreased risk of HS in the group of ≤50 years old, respectively, and the *TNFRSF11B* rs2073618 variants is a risk factor in the susceptibility of HS in the >50 year group in Chinese population for the first time. The association between *TNFRSF11B* rs2073617 and the risk of HS was not found, irrespective of age stratification. However, further studies in other populations are needed to be warranted.

## Conflict of Interest

None declared.
